# Effects of bicuspid extractions and incisor retraction on upper airway of Asian adults and late adolescents: A systematic review

**DOI:** 10.1111/joor.12854

**Published:** 2019-08-01

**Authors:** Jing Hao Ng, Yi Lin Song, Adrian U. J. Yap

**Affiliations:** ^1^ National Dental Centre Singapore Singapore Singapore; ^2^ Department of Dentistry, Ng Teng Fong General Hospital National University Health System Singapore Singapore; ^3^ Faculty of Dentistry National University of Singapore Singapore Singapore

**Keywords:** airway, breathing, obstructive sleep apnoea, orthodontics, review, tooth extraction

## Abstract

**Objectives:**

This systematic review aimed to assess the effects of bicuspid extractions and incisor retraction on airway dimension, hyoid position and breathing of adults and late adolescents.

**Methods:**

The review was conducted according to PRISMA guidelines. Eight databases including PubMed, EMBASE, Web of Science and Scopus were searched to August 2018. Minimum age of participants was 16 years. The intervention was dual‐arch bicuspid extractions with incisor retraction. Outcomes were airway dimension, hyoid position and breathing assessment.

**Results:**

All nine publications meeting inclusion criteria were from Asia. They were divided into three Asian subregions. All East Asian lateral cephalometric studies reported anteroposterior airway narrowing at the oropharynx and sometimes the hypopharynx. However, the narrowing was small, comparable to measurement errors, and highly variable. Two out of three East Asian computed tomography (CT) studies described reductions in airway dimensions. The single functional breathing study showed increased simulated flow resistance after incisor retraction in East Asians. South Asian studies had mixed findings, with some reporting significant airway narrowing. The single study from West Asia found no significant airway or hyoid changes.

**Conclusions:**

Airway response to bicuspid extractions and incisor retraction varied substantially when assessed with cephalometry. CT measurements present larger effect sizes and smaller variations, providing stronger evidence of airway narrowing. Orthodontic extractions for incisor retraction may be more frequently indicated in Asia, and East Asians seem particularly susceptible to airway narrowing and postero‐inferior hyoid movement with incisor retraction. Better designed CT studies are needed for confirmation due to small effect size and large variability.

## 1 INTRODUCTION

Obstructive sleep apnoea (OSA) is a condition characterised by repeated collapse of the upper airway during sleep, leading to oxygen desaturations, persistent respiratory effort, arousals and sleep fragmentation.^1^ It is defined by the occurrence of daytime sleepiness, loud snoring, witnessed breathing interruptions or awakenings due to gasping or choking in the presence of at least five obstructive respiratory events per hour of sleep (apnoea–hypopnea index [AHI] > 5).^2^ The prevalence of moderate to severe OSA with AHI ≥ 15 is as high as 30%‐50%, with the majority of subjects not diagnosed.^3^, ^4^, ^5^ Severe OSA is associated with increased mortality, cardiovascular diseases, stroke, diabetes, motor vehicle accidents, cognitive impairments and reduced quality of life.^6^

Obstructive sleep apnoea is a heterogeneous disorder, with obesity, age, oropharyngeal and facial anatomy,^7^ as well as non‐anatomical and functional factors such as neuromuscular feedback and airway collapsibility playing pathogenic roles in OSA.^8^, ^9^, ^10^ Anatomic factors are important contributors and have been correlated to OSA severity.^11^, ^12^, ^13^, ^14^ Some clinicians have suggested that tooth extractions predispose patients to OSA. The proposed mechanism is a reduced arch depth in the sagittal plane resulting in decreased oral cavity volume and posterior displacement of the tongue and soft palate. The reduction in arch depth may be more significant in certain skeletal types, particularly Class II subtypes, and the decrease in airway space may lead to possible aggravation of snoring and OSA.^15^, ^16^ Reopening of closed orthodontic extraction spaces was even recommended to resolve OSA.^17^ Clinically, Fukuda et al^18^ found higher AHI in orthodontic extraction patients compared with matched untreated controls. Conversely, Larsen et al^19^ found no difference in OSA prevalence between patients with orthodontic extractions and matched controls. As it is difficult to link orthodontic treatment performed in adolescence^20^ with development of OSA in later adulthood,^21^, ^22^, ^23^ changes in airway anatomy are often used as a proxy for OSA risk, as OSA severity is correlated to anteroposterior (A‐P) airway dimension, cross‐sectional airway area (CSA), pharyngeal airway length, hyoid bone position and airway resistance.^11^, ^12^, ^13^, ^14^

Decrease in airway space^24^, ^25^ and changes in hyoid bone position^25^, ^26^ after orthodontic extractions have been reported. Conversely, other studies have found no change in airway space^27^, ^28^, ^29^ and hyoid position^30^ after orthodontic extractions. The lack of consensus could be attributed to differences in patient age and extraction indications.^31^, ^32^ Airway effects from orthodontic extractions in growing patients may be ameliorated by pharyngeal growth.^27^, ^28^, ^32^ Different orthodontic mechanics can also have differing airway effects.^29^, ^31^, ^32^ A prior systematic review^31^ investigating the effect of teeth extractions on airway dimensions found a limited number of studies and great heterogeneity in patient groups and orthodontic indications. For greater clarity, this systematic review will focus on the subset of orthodontic extractions with upper and lower incisor retraction in adults and late adolescents.

The objectives of this systematic review were thus to investigate the effects of bicuspid extraction and orthodontic incisor retraction in adults and late adolescents on:

1. Linear, cross‐sectional and volumetric measurements of posterior airway space;
2. Horizontal and vertical position of hyoid bone;
3. Functional measures of breathing.

## 2 MATERIALS AND METHODS

This systematic review was reported according to the Preferred Reporting Items for Systematic Reviews and Meta‐Analyses (PRISMA) guidelinesa
http://prisma-statement.org/documents/PRISMA%25202009%2520checklist.pdf
.^33^ The review was registered with the PROSPERO database (PROSPERO 2018 CRD42018102318)b
http://www.crd.york.ac.uk/PROSPERO/display_record.php?ID=CRD42018102318
.

### 2.1 Search Strategy

Eight databases were systematically searched from their inception up to August 2018 (using the search terms detailed in Table 1). They included PubMed, EMBASE, Web of Science, Scopus, Cochrane Central Register of Controlled Trials, Cochrane Database of Systematic Reviews, Google Scholar and WorldWideScience. A limited “grey” literature search was conducted via the latter two databases. The reference and citation lists of all pertinent publications including systematic reviews^31^ were manually searched for additional eligible studies. The search was independently conducted by two authors (NJH and SYL).

**Table 1:** Search strategy and outcomes

	Database/ Aggregator	Search strategy used	Extent of search	Citations found
1	PubMed	("tooth extraction" [mesh] OR ((tooth OR teeth OR premolar* OR bicuspid* OR orthod*) AND extract*)) AND airway	In all fields	186
2	EMBASE	('tooth extraction'/exp OR (('tooth'/exp OR tooth OR 'teeth'/exp OR teeth OR premolar* OR bicuspid* OR orthod*) AND extract*)) AND ('airway'/exp OR airway)	In all fields	299
3	Web of Science	TOPIC: ((tooth OR teeth OR premolar* or bicuspid* or orthod* AND extract*) AND airway) Refined by: WEB OF SCIENCE CATEGORIES: ( DENTISTRY ORAL SURGERY MEDICINE OR SURGERY OR MEDICINE GENERAL INTERNAL OR RESPIRATORY SYSTEM OR OTORHINOLARYNGOLOGY) Timespan: All years. Indexes: SCI‐EXPANDED, SSCI, A&HCI, CPCI‐S, CPCI‐SSH, ESCI.	In the topic	302
4	Scopus http://www.scopus.com	TITLE‐ABS‐KEY ( ( ( "tooth extraction" OR ( ( tooth OR teeth OR premolar* OR bicuspid* OR orthod*) AND extract*)) AND airway)) AND (LIMIT‐TO ( DOCTYPE, "ar") OR LIMIT‐TO ( DOCTYPE, "re")) AND ( LIMIT‐TO ( SUBJAREA, "MEDI") OR LIMIT‐TO ( SUBJAREA, "DENT"))	In title, abstract, keywords	231
5	Cochrane Central Register of Controlled Trials (CENTRAL)	((tooth OR teeth OR premolar* OR bicuspid* OR orthod*) AND extract*) AND airway	All Text (Word variations have been searched)	18
6	Cochrane Database of Systematic Reviews	((tooth OR teeth OR premolar* OR bicuspid* OR orthod*) AND extract*) AND airway	All Text (Word variations have been searched)	28
7	Google Scholar	allintitle: ((tooth OR teeth OR premolar OR premolars OR bicuspid OR bicuspids OR orthodontic OR orthodontics) (extract OR extraction OR extractions)) airway	All in title	24
8	World Wide Science worldwidescience.org	((tooth OR teeth OR premolar* OR bicuspid* OR orthod*) AND extract*) AND airway	Full Record (English)	564
	Sum			1652

### 2.2 Selection criteria

The following inclusion and exclusion criteria were defined *a priori*.

#### 2.2.1 Study types

Randomised clinical trials, quasi‐experimental studies, prospective and retrospective cohort studies, case–control studies and case series were included, while all other study designs were excluded.

#### 2.2.2 Study language

Studies were restricted to those reported in the English language.

#### 2.2.3 Study participants

Studies where the subjects were above 16 years old were included. All races, genders, malocclusions, vertical and horizontal skeletal subtypes were included.

#### 2.2.4 Study intervention

The intervention was orthodontic treatment with dual‐arch bicuspid extractions plus upper and lower incisor retraction. The intervention must be accompanied by examination with two‐dimensional (2D) or three‐dimensional (3D) radiographic examination before and after orthodontic treatment or retraction of incisors. Studies with single‐arch extractions or extractions without mention or measurement of incisor retraction were excluded. Studies with subjects undergoing growth modification or orthognathic surgery were also excluded, as these may produce airway changes independent of the extraction treatment.^34^, ^35^

#### 2.2.5 Study comparison

Treated subjects were compared with untreated controls or non‐extraction controls where applicable.

#### 2.2.6 Study outcome measures

The outcome variables evaluated were as follows:

- Linear upper airway measurements.
- Cross‐sectional upper airway changes.
- Volumetric upper airway changes.
- Vertical and horizontal changes in hyoid bone position.
- Functional assessment of breathing.

### 2.3 Data collection and synthesis

The titles and abstracts of identified studies were screened independently by two authors (NJH and SYL), followed by an independent checking of their full texts for eligibility by both authors. Any conflicts at either stage were resolved by full‐text screening and moderation by a third author (YAU). Final decisions were made after consensus was reached.

#### 2.3.1 Data extraction and management

Data extraction was performed independently by two authors (NJH and SYL) using pre‐determined data extraction forms. Discrepancies in data extraction between the two authors were likewise resolved by the third author (YAU). Corresponding authors were contacted by email when clarifications on study design were required or when there was incomplete reporting of results.

#### 2.3.2 Assessment of methodological quality

The Joanna Briggs Institute's Critical Appraisal Checklist was used to assess methodological quality of the selected studies (Table 2). This was assessed independently by two authors (NJH and SYL), and conflicts between them were resolved by the third author (YAU).

**Table 2:** Assessment of methodological quality

	West Asian			South Asian			East Asian		
	Al Maaitah 2012^30^	Bhatia 2016^52^	Nagmode 2017^53^	Patel 2017^51^	Keum 2017^50^	Wang 2012^25^	Zhang 2015^49^	Chen 2012^26^	Zheng 2017^54^
Were there clear criteria for inclusion in the case series?	Y	Y	Y	Y	Y	Y	Y	N	Y
Was the condition measured in a standard, reliable way for all participants included in the case series?	Y	Y	Y	Y	Y	Y	Y	Y	Y
Were valid methods used for identification of the condition for all participants included in the case series?	Y	Y	Y	Y	Y	Y	Y	Y	Y
Did the case series have consecutive inclusion of participants?	U	U	U	N^a^	Y	U	U	U	N
Did the case series have complete inclusion of participants?	U	Y	U	N^a^	Y	Y	U	U	N
Was there clear reporting of the demographics of the participants in the study?	N	N	N	Y^a^	N	N	N	N	N
Was there clear reporting of clinical information of the participants?	Y	Y	Y	Y^a^	N	Y	Y	N	Y
Were the outcomes or follow‐up results of cases clearly reported?	Y	Y	Y	Y	N	Y	Y	Y	Y
Was there clear reporting of the presenting site(s)/clinic(s) demographic information?	Y	N	Y	Y	Y	Y	Y	U	Y
Was statistical analysis appropriate?	Y	Y	Y	Y	Y	Y	Y	Y	Y

Abbreviations: N, no; NA, not applicable; U, unclear; Y, yes.

a Author correspondence.

## 3 RESULTS

### 3.1 Yield of search

The search strategy yielded a total of 1652 articles and abstracts, of which 441 were duplicates. Screening of the titles and abstracts of the remaining 1211 articles resulted in 24 articles selected for full‐text assessment. However, the full texts of three articles were inaccessible.^36^, ^37^, ^38^ After full‐text appraisal, 12 articles were excluded due to the following reasons:

1. Full text not in English.^39^, ^40^, ^41^, ^42^
2. Treatment group below 16 years old.^24^, ^27^, ^43^, ^44^
3. Single‐arch extraction.^45^, ^46^
4. Incisor retraction not uniformly applied.^29^
5. Unclear inclusion criteria, no email response from authors.^47^

**Figure 1:**
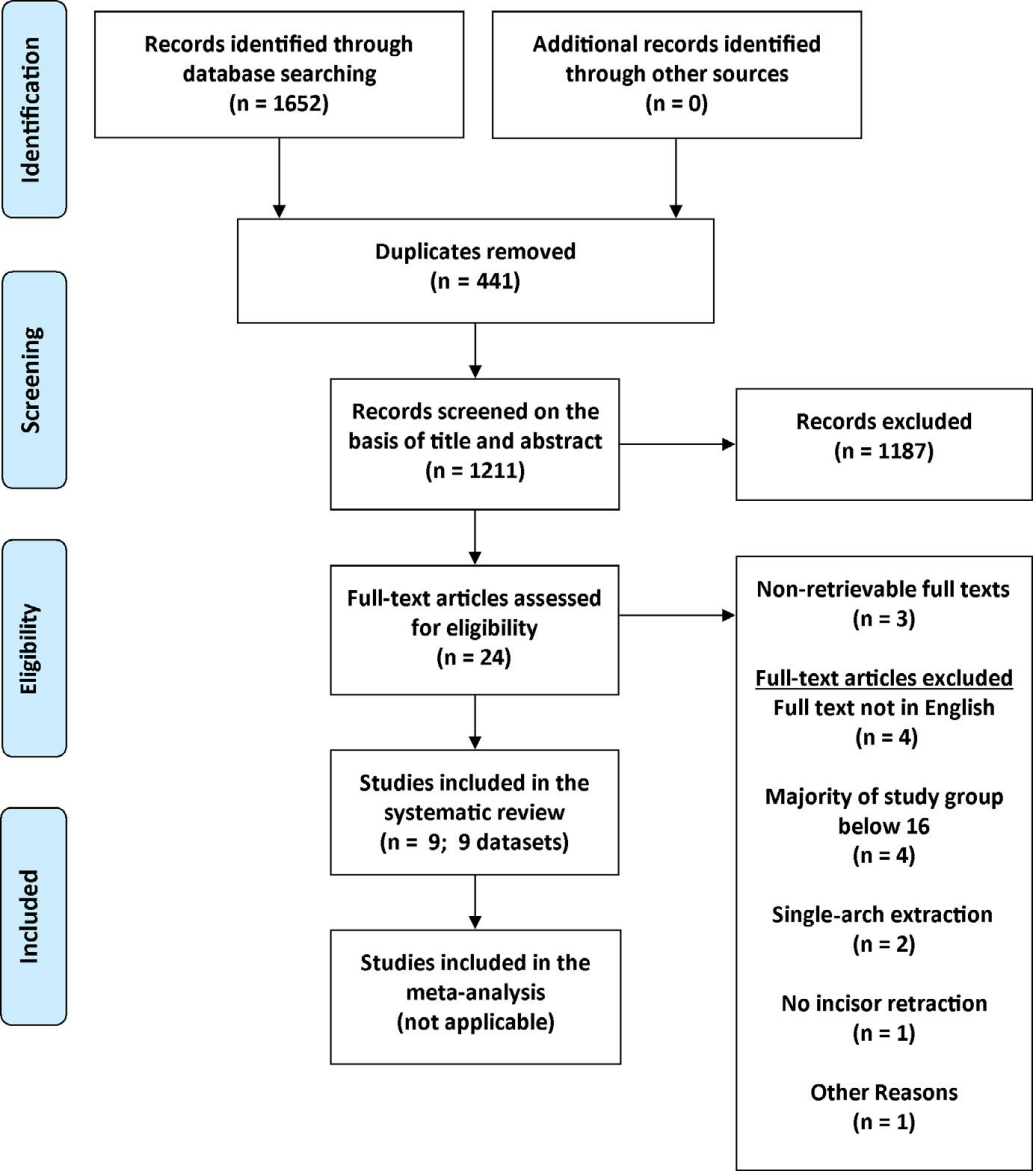
PRISMA 2009 flow diagrams [Colour figure can be viewed at http://www.wileyonlinelibrary.com/]

Nine eligible articles were selected for this systematic review and narrative synthesis (Figure 1).

### 3.2 Study characteristics

The selected studies were all from Asia and were divided by subregions based on United Nations’ classification of macrogeographic subregions.^48^ The studies were further organised into lateral cephalometric and computed tomography (CT) studies, with one study using both CT and a CT‐derived midsagittal lateral cephalometric image for airway measurements.^49^ All studies were uncontrolled before–after case series. Two of the studies^50^, ^51^ reported data from multiple patient groups, from which only the study group with incisor retraction was used in the review. Keum et al^50^ studied both an incisor retraction group and a mandibular setback group. Patel et al^51^ reported both a Class I incisor retraction group and a Class II division 1 extraction group. Zhang et al^49^ attempted to use a control group but only made a cross‐sectional comparison of airway sizes with the treatment group at the post‐treatment time point. As there was no assessment of changes in airway dimensions for the control group over the treatment duration, it was deemed to be a case series. Additional information about the studies is shown in Table 3. Outcome measures and landmarks used in lateral cephalometric studies are shown in Table 4.

**Table 3:** Characteristics of selected studies

Subregion	Study	Study design	Imaging modality	Number of patients (Male, Female)	Minimum age	Skeletal maturity	Weight/ Body Mass Index (BMI)	Horizontal skeletal classification	Vertical skeletal classification
West Asia (Jordan)	Al Maaitah 2012^30^	Retrospective case series	Lateral cephalogram	40 (13M, 27F)	18 y	Not mentioned	Not mentioned	Not mentioned. Average ANB 3.55 degrees (SD 2.06)	Not mentioned. Average MMPA 28.19 degrees (SD 4.88)
South Asia (India)	Bhatia 2016^52^	Retrospective case series	Lateral cephalogram	22 (9M, 13F)	17 y	Not mentioned	Not mentioned	Skeletal class I	Not mentioned. Average FMA 29.50 degrees (SD 5.56)
South Asia (India)	Nagmode 2017^53^	Retrospective case series	Lateral cephalogram	30 (no gender breakdown)	16 y	Not mentioned	Not mentioned	Not mentioned. Average ANB 3.3 degrees (SD 1.96)	Not mentioned. Average SN‐Mandibular Plane 29.5 degrees (SD 6.86)
South Asia (India)	Patel 2017^51^	Retrospective case series	Lateral cephalogram	20 (6M, 14F)	16 y/Completed skeletal growth according to skeletal growth indicators^a^.	Cervical growth maturation staging and hand‐wrist radiographs^a^	Not mentioned	Not mentioned. Average ANB 3.88 degrees (SD 1.83)	Not mentioned. Average FMA 27.70 degrees (SD 7.96)
East Asia (South Korea)	Keum 2017^50^	Retrospective case series	Lateral cephalogram	33 (17M, 16F)	17 y	Not mentioned	Not mentioned	Not mentioned. Average ANB 3.5 degrees (SD 3.06)	Not mentioned. Average FMA 28.89 degrees (SD 6.14)
East Asia (China)	Wang 2012^25^	Retrospective case series	Lateral cephalogram	44 (8M, 36F)	16 y	Not mentioned	BMI within normal limits (18.5‐23.9)	Skeletal class I	Non‐hyperdivergent group FHMP < 30.5 degrees. Hyperdivergent group FHMP > 30.5 degrees. Combined for analysis.
East Asia (China)	Zhang 2015^49^	Retrospective case series	Cone beam computed tomography + CT‐derived Lateral cephalogram from mid‐sagittal plane	18 (5M, 13F)	18 y	Not mentioned	BMI within normal limits 20.33 (SD 1.77)	Skeletal class II with ANB more than 4.7 degrees	Hyperdivergent with MPSN more than 37.7 degrees
East Asia (China)	Chen 2012^26^	Prospective case series	Multislice computed tomography	30 (no gender breakdown)	Not mentioned. Inclusion criteria: Adult patients	Not mentioned	Not mentioned	Not mentioned	Not mentioned
East Asia (China)	Zheng 2017^54^	Prospective case series	Cone beam computed tomography	30 (11M, 19F)	18 y	Not mentioned	BMI 20.56 (SD 1.48)	Not mentioned	Not mentioned

Subregion	Study	Dental classification	Crowding/ Spacing	Upper extraction	Lower Extraction	Retraction plan and mechanics	Anchorage support
West Asia (Jordan)	Al Maaitah 2012^30^	Class I molar + Bimaxillary proclination	No crowding or spacing at the start of treatment	First premolars	First premolars	Reduce incisal proclination and lip procumbency.	Not mentioned
South Asia (India)	Bhatia 2016^52^	Class I canine and molar + Bimaxillary protrusion	Well‐aligned arches with no or minimal crowding	First premolars	First premolars	Group A anchorage—retraction of anterior teeth as standard care of treatment.	Vertical pull headgear + banding of second molars + Nance button OR Transpalatal arch
South Asia (India)	Nagmode 2017^53^	Class I canine, premolar and molar + Bimaxillary protrusion	Well‐aligned arches with no or minimal crowding	First premolars	First premolars	Maximum anchorage with maximal retraction of anterior teeth.	Not mentioned
South Asia (India)	Patel 2017^51^	Class I molar + Class I Bimaxillary protrusion	Not mentioned	First premolars	First premolars^a^	Significant upper and lower anterior retraction^a^	Not mentioned
East Asia (South Korea)	Keum 2017^50^	Not Mentioned	Not mentioned	One premolar per quadrant, tooth not specified.	One premolar per quadrant, tooth not specified.	More than 5 mm bodily retraction of lower incisors.	Miniscrews placed between second premolar and first molar
East Asia (China)	Wang 2012^25^	Class I canine, premolar and molar + Bimaxillary protrusion	Well‐aligned arches with no or minimal crowding	First premolars	First premolars	Maximum anchorage with maximal retraction of anterior teeth.	Headgear or miniscrew
East Asia (China)	Zhang 2015^49^	Class II canine and molar	Upper mild crowding. Lower mild to moderate crowding.	First premolars	First premolars/ Second premolars (6 out of 18 patients had second premolars removed)	Maximum anchorage.	Miniscrew
East Asia (China)	Chen 2012^26^	Bimaxillary dentoalveolar protrusion	Not mentioned	First premolars	First premolars	Large incisor retraction. Retraction and intrusion of upper incisors.	Miniscrew
East Asia (China)	Zheng 2017^54^	Class I Bimaxillary dentoalveolar protrusion	Not mentioned	First premolars	First premolars	Maximum anchorage for retraction and intrusion of anterior teeth.	Miniscrew

a Author correspondence.

**Table 4:** Lateral cephalometric measures and landmarks

	Description	Studies
Upper airway		
E‐IPW/mm	Distance between E and IPW	Keum 2017
PNS‐Ad1/mm	Distance between PNS and Ad1	Bhatia 2016, Wang 2012
PNS‐R/mm	Distance between PNS and R	Bhatia 2016, Wang 2012, Zhang 2015
PNS‐SPW/mm	Distance between PNS and SPW	Keum 2017
PNS‐UPW/mm	Distance between PNS and UPW	Zhang 2015
SPP‐SPPW/mm	Distance between SPP and SPPW	Bhatia 2016, Wang 2012, Zhang 2015
TB‐TPPW/mm	Distance between TB and TPPW	Bhatia 2016, Nagmode 2017, Patel 2017, Wang 2012, Zhang 2015
U‐MPW/mm	Distance between U and MPW	Bhatia 2016, Keum 2017, Wang 2012, Zhang 2015
VAL/mm	Vertical airway length (distance between PNS and V)	Bhatia 2016, Nagmode 2017, Patel 2017, Wang 2012
V‐LPW/mm	Distance between V and LPW	Bhatia 2016, Wang 2012, Zhang 2015
PAS/mm	Width of the airway space along the Go‐B line	Zhang 2015
SPAS/mm	Width of airway behind soft palate along line which is parallel to Go‐B line	Nagmode 2017, Patel 2017
MAS/mm	Width of airway along parallel line to Go‐B line through P	Nagmode 2017, Patel 2017
McNamara's upper pharynx dimension/mm	Minimum distance between the upper soft palate and the nearest point on the posterior pharynx wall	Nagmode 2017
McNamara's lower pharynx dimension/mm	Minimum distance between the point where the posterior tongue contour crosses the mandible and the nearest point on the posterior pharynx wall	Nagmode 2017
Upper airway thickness/mm	Distance between PNS and the nearest adenoid tissue measured through a perpendicular line to S‐Ba from PNS	Nagmode 2017
Lower airway thickness/mm	Distance between PNS and the nearest adenoid tissue through PNS‐Ba line	Nagmode 2017
Hyoid position		
C3H/mm	Distance between H and C3	Bhatia 2016, Nagmode 2017, Patel 2017, Wang 2012, Zhang 2015
HH1/mm	Perpendicular distance from hyoid bone to the line connecting C3 and RGN	Bhatia 2016, Nagmode 2017, Wang 2012
H‐HRP/mm	Distance from point H to HRP (horizontal reference plane—the Frankfort horizontal plane)	Keum 2017, Zhang 2015
H‐MP/mm	Perpendicular distance from H to mandibular plane (MP)	Nagmode 2017, Zhang 2015
H‐RGN/mm	Distance between H and RGN	Bhatia 2016, Nagmode 2017, Wang 2012, Zhang 2015
H‐VRP/mm	Distance from point H to VRP (vertical reference plane—passes through S, perpendicular to HRP)	Keum 2017
SH/mm	Distance between S and H	Bhatia 2016, Patel 2017, Wang 2012
LANDMARKS		
AD1	Point of intersection of posterior pharyngeal wall and line Ptm‐Ba	
B	The deepest point in the curvature of the mandibular alveolar process	
Ba	Basion	
C3	Most anteroinferior point of the third vertebra	
E	Tip of the epiglottis	
Go	Gonion	
H	Most superior and anterior point of hyoid bone	
H1	Foot point of perpendicular line from RGN to C3	
Hor	Most inferior point of spheno‐occipital synchondrosis	
IPW	Inferior pharyngeal wall, point of intersection of the posterior pharyngeal wall and perpendicular line drawn from the E	
LPW	Foot point of perpendicular line from point V to posterior pharyngeal wall	
MPW	Foot point of perpendicular line from point U to posterior pharyngeal wall	
PNS	Posterior nasal spine	
Ptm	Pterygomaxillary fissure	
R	Point of intersection of line from Hor to PNS and posterior pharyngeal wall	
RGN	Most protrusive point of retrognathion	
S	Sella	
SPP	Point of intersection of line from soft palate centre perpendicular to posterior pharyngeal wall and posterior margin of soft palate	
SPPW	Point of intersection of line from soft palate centre perpendicular to posterior pharyngeal wall	
SPW	Superior pharyngeal wall	
TB	Point of intersection of base of the tongue and extension of line B‐Go	
TPPW	Point of intersection of posterior pharyngeal wall and extension of line B‐Go	
U	Tip of the uvula	
UPW	Point locates at the intersection between posterior pharyngeal wall and PNS‐Ba line	

### 3.3 Airway changes

#### 3.3.1 Linear changes

All three East Asian lateral cephalometric studies reported linear airway narrowing in the A‐P dimension with incisor retraction. This was reported at the retropalatal,^25^, ^49^, ^50^ retroglossal^25^, ^49^ and hypopharyngeal levels.^25^, ^49^ No changes were seen at the level of the nasopharynx. Airway length was measured by only one study and was found to be increased after incisor retraction.^25^

Of the three South Asian studies, one reported no significant changes in airway dimensions^51^ while two studies showed linear dimensional reduction at the retropalatal^52^ and retroglossal levels.^52^, ^53^ Nasopharyngeal airway dimensional increase was reported by one study and attributed to lymphoid mass regression.^53^ No significant change in airway length was found.

The West Asian study^30^ found no significant change in airway dimensions from anterior retraction and arch dimension reduction in the treatment of bimaxillary proclination.

#### 3.3.2 Cross‐sectional changes

All three CT studies^26^, ^49^, ^54^ used different methods of measuring airway changes and slightly different landmarks and planes to divide the airway. Chen et al^26^ reported a decrease in mean CSA at the retropalatal, retroglossal and hypopharyngeal levels, with no significant change in mean CSA at the nasopharyngeal level. Zheng et al^54^ stated that the minimum CSA for the whole airway was significantly decreased. In addition, the location of minimum CSA of the airway moved from the hypopharynx pre‐treatment to the oropharynx post‐treatment. In contrast to Chen et al^26^ and Zheng et al,^54^ Zhang et al^49^ found no change in the CSA from incisor retraction, but reported a cross‐sectional shape change with decreased A‐P dimension but increased lateral width, which maintained the overall CSA for the airway.

#### 3.3.3 Volumetric changes

Two studies reported airway volume changes after incisor retraction. Zheng et al^54^ found a significant reduction in the oropharyngeal airway volume, whereas Zhang et al^49^ reported no significant change in volume at each level of the airway and in the total airway volume.

#### 3.3.4 Airway changes in relation to incisor retraction

Changes in airway dimension with respect to incisor retraction were investigated by four out of the five East Asian studies^25^, ^26^, ^50^, ^54^ and one of the South Asian studies.^52^

For East Asian subjects, Wang et al^25^ reported that the decrease in linear dimensions of the retropalatal and retroglossal airway was correlated to lower incisor retraction distance. No correlations, however, were found by Keum et al.^50^ Both incisor retraction by uprighting^25^, ^49^ and bodily retraction^50^ resulted in reduction of linear airway dimensions. With regard to CT studies, Chen et al^26^ reported that CSA decrease was correlated to upper incisor tip retraction. Similarly, Zheng et al^54^ reported that the increase in flow resistance of the entire airway as well as at the oropharyngeal and hypopharyngeal levels was correlated with upper incisor tip retraction. However, both these CT studies did not measure the amount of lower incisor retraction.

In South Asian subjects, Bhatia et al^52^ reported that linear dimension reduction at both the retropalatal and retroglossal levels was significantly correlated with lower incisor retraction distance.

### 3.4 Hyoid changes

One out of the five East Asian studies did not study hyoid bone changes.^54^ Of the four that did, three studies reported an inferior movement of the hyoid,^25^, ^26^, ^50^ while two studies reported a backward movement of the hyoid.^25^, ^26^ Zhang et al,^49^ however, found no significant horizontal or vertical hyoid movement.

None of the South Asian studies reported significant vertical hyoid bone movements. In the horizontal plane, Bhatia et al^52^ did not express hyoid movement clearly and no clarification was received from the authors. The remaining two South Asian studies did not report significant horizontal hyoid movements.

The West Asian study^30^ found no significant change in hyoid bone position.

### 3.5 Functional measures of breathing

Flow resistance was reported by Zheng et al^54^ and was ascertained by computational fluid dynamics on 3D reconstructed airway models. There was no significant change in nasopharynx resistance. Airflow resistance was significantly increased by 87.43% at the oropharynx, 27.14% at the hypopharynx, and 78.14% across the entire airway with incisor retraction.

Changes in airway dimension, hyoid bone position and functional breathing are summarised in Table 5.

**Table 5:** Changes in airway, hyoid position and functional breathing

Subregion	Study	Total airway ‐ vertical length				Nasopharynx				Oropharynx retropalatal				Oropharynx retroglossal			
		Metric	Change ±	SD	% Change ±	Metric	Change ±	SD	% Change ±	Metric	Change ±	SD	% Change ±	Metric	Change ±	**SD**	% Change ±
West Asia (Jordan)	Al Maaitah 2012^30^	Not significant				Not significant				Not significant				Not significant			
South Asia (India)	Bhatia 2016^52^	Not significant				Not significant				SPP‐SPPW	−2.6 mm	2.77 mm	−16.72%	TB‐TPPW	−2.65 mm	1.47 mm	−19.56%
										U‐MPW	−2.85 mm	1.84 mm	−22.27%				
South Asia (India)	Nagmode 2017^53^	Not significant				Upper Airway Thickness	+1.20 mm	Not Reported	+4.31%	Not significant				TB‐TPPW	−0.40 mm	Not Reported	−4.94%
South Asia (India)	Patel 2017^51^	Not significant				Not measured				Not significant				Not significant			
East Asia (South Korea)	Keum 2017^50^	Not measured				Not significant				U‐MPW	−1.15 mm	1.17 mm	−10.39%	Not significant			
East Asia (China)	Wang 2012^25^	VAL (PNS‐V)	+1.00 mm	3.03 mm	+1.71%	Not significant				SPP‐SPPW	−0.56 mm	1.48 mm	−4.07%	TB‐TPPW	−1.63 mm	1.80 mm	−13.71%
										U‐MPW	−0.85 mm	1.77 mm	−7.88%				
East Asia (China)	Zhang 2015^49^	Not Significant				PNS‐R	Not Significant			SPP‐SPPW	−1.36 mm	1.91 mm	−10.44%	TB‐TPPW	−1.80 mm	2.39 mm	−15.69%
						PNS‐UPW	Not Significant			U‐MPW	−1.07 mm	1.93 mm	−9.47%				
						CSA PNS‐R	+20.66 mm^2^	25.28 mm^2^	+4.30%	CSA SPP‐SPPW	Not Significant			CSA TB‐TPPW	Not significant		
						CSA PNS‐UPW	Not significant			CSA U‐MPW	Not significant						
						Volume	Not Significant			Volume^a^	Not Significant			a			
East Asia (China)	Chen 2012^26^	Not measured				Not significant				Mean CSA	Not reported	7.89%	−21.02%	Mean CSA	Not reported	13.51%	−25.81%

		Total Airway ‐ Other Measures				Nasopharynx				Oropharynx				
		Metric	Change ±	SD	% Change ±	Metric	Change ±	SD	% Change ±	Metric	Change ±	SD	% Change ±	
East Asia (China)	Zheng 2017^54^	Minimum CSA	−70 mm^2^	Not reported	−31.67%	Not significant				Volume^b^	−310 mm^3^	Not reported	−24.92%	b
		Flow resistance	+25.85 Pa	Not reported	+78.14%					Flow Resistance^b^	+17.46 Pa	Not reported	+87.43%	

Subregion	Study	Hypopharynx				Hyoid horizontal				Hyoid vertical			
		Metric	Change ±	SD	% Change ±	Metric	Change ±	SD	% Change ±	Metric	Change ±	SD	% Change ±
West Asia (Jordan)	Al Maaitah 2012^30^	Not measured				Not significant				Not significant			
South Asia (India)	Bhatia 2016^52^	Not Significant				Unclear results. Author contacted but no clarification received.				Not significant			
South Asia (India)	Nagmode 2017^53^	Not measured				Not significant				Not significant			
South Asia (India)	Patel 2017^51^	Not significant				Not significant				Not significant			
East Asia (South Korea)	Keum 2017^50^	Not significant				Not significant				H‐HRP	+2.46 mm	3.70 mm	+2.61%
East Asia (China)	Wang 2012^25^	V‐LPW	−1.54 mm	2.90 mm	−9.52%	C3‐H	−0.88 mm	2.32 mm	−2.73%	S‐H	+1.24 mm	3.24 mm	+1.26%
						H‐RGN	Not significant			H‐H1	Not significant		
East Asia (China)	Zhang 2015^49^	V‐LPW	−0.94 mm	1.53 mm	−6.15%	Not significant				Not significant			
		CSA V‐LPW	Not significant			Not significant				Not significant			
		Volume	Not significant			Not significant				Not significant			
East Asia (China)	Chen 2012^26^	Mean CSA	Not reported	5.51%	−38.19%	X‐Hm	−2.96 mm	0.54 mm	Not Reported	Y‐Hm	−9.87 mm	2.92 mm	Not Reported
East Asia (China)	Zheng 2017^54^	Flow Resistance	+6.46 Pa	Not reported	+27.14%	Not measured				Not measured			

a Zhang 2015—volumes reported for oropharynx, not divided into retropalatal and retroglossal regions.

b Zheng 2017— volume and flow resistance reported for oropharynx, not divided into retropalatal and retroglossal regions.

## 4 DISCUSSION

### 4.1 General remarks

The effects of bicuspid extraction and incisor retraction on airway dimension, hyoid position and breathing of adults and late adolescents were systematically reviewed in the present work. The PRISMA guideline was adopted to improve reporting transparency.^33^ The review was restricted to those published in English due to journal access and language literacy issues. All the selected studies were case series, and the Joanna Briggs Institute's (JBI) critical appraisal checklistcAvailable at: https://wiki.joannabriggs.org/display/MANUAL/Appendix+7.3+Critical+appraisal+checklists+for+case+series. Accessed January 2019. for case series was employed to assess the methodological quality of the studies. The JBI is an international partnership behind the creation, transfer and utilisation of evidence‐based healthcare practices aimed at improving care outcomes.

#### 4.1.1 Age and growth status

Based on the preliminary data search for adult studies, for the purposes of this review, the cut‐off age was extended to late adolescence and set at 16 years old as several studies had adult patient groups defined as 16 years old,^25^, ^51^, ^53^ 17 years old^50^, ^52^ or 18 years old.^30^, ^49^, ^54^ None of the studies reported growth assessment prior to commencing orthodontic treatment, but one author clarified that cervical growth maturation staging and hand‐wrist radiographs were used for growth assessment.^51^

Skeletal growth has been reported to cease at an average age of 17.5 years for females and 19.2 years for males.^55^ For the upper airway, the major growth phases have been reported to be from 0 to 5 years, 6 to 9 years and 12 to 16 years old.^56^, ^57^ Quiescence of airway growth has been noted from 9 to 12 years and 15 to 18 years.^56^ However, airway size and length have also been reported to increase until age 20.^58^ The possible continued pharyngeal airway growth after 16 years old could have mitigated the amount of airway narrowing caused by incisor retraction. Similarly, Taylor et al^56^ reported that the hyoid bone continues to descend and moves slightly anteriorly up to age 18. This could have confounded the findings on hyoid position in studies with younger subjects. As all the studied papers did not have control groups, the effect of continued growth on hyoid position and airway dimensions cannot be ruled out.

### 4.2 Changes in airway and hyoid position

#### 4.2.1 By Asian subregion

All except one East Asian study reported airway dimensional reduction at the oropharynx and sometimes the hypopharynx as well as inferior and/or posterior movement of the hyoid bone after incisor retraction. However, the stated linear airway narrowing and hyoid bone movements in lateral cephalometric studies were small and comparable to the estimated 1.0 mm to 1.55 mm error of cephalometric airway measurements^59^, ^60^, ^61^ and the 1.02 mm to 2.16 mm error for hyoid measurements.^59^

The single contrasting study by Zhang et al^49^ found no significant cross‐sectional and volumetric airway changes and no significant hyoid movements. Although the study found significant A‐P airway reduction that was also reported in the other East Asian lateral cephalometric studies, the A‐P reduction was offset by a transverse widening that maintained airway CSA and volume. The compensatory shape change was not observed in the other two CT studies. The differences could be because:

1. Zhang's study was the only one conducted on skeletal Class II hyperdivergent patients.
2. In contrast to the upper and lower first premolars extraction pattern in the other studies, orthodontic intervention in Zhang's study included either lower first or second premolar extractions, which may necessitate different orthodontic mechanics for space closure.

As previous authors have suspected that airway narrowing from incisor retraction may be more significant in patients with Class II skeletal bases^16^ and because the hyperdivergent subtype is predisposed to OSA,^14^ the adaptive cross‐sectional shape change in response to incisor retraction reported by Zhang et al 49 was unexpected. Further studies in hyperdivergent skeletal Class II patients are warranted.

Unlike the East Asian studies, all three South Asian studies were conducted on fairly similar study populations who received comparable orthodontic interventions. However, results were not uniform, with one study finding no significant airway narrowing and two studies describing significant oropharyngeal narrowing after incisor retraction. Of the two studies with significant airway narrowing, one reported an oropharyngeal narrowing of 0.40 mm, well within the error of cephalometric airway measurements.^59^, ^60^, ^61^

The single West Asian study^30^ found no significant change in airway dimensions or hyoid bone position.

#### 4.2.2 Hyoid measurements

The majority of studies use H‐MP, HH1 and H‐RGN to measure vertical and horizontal changes in the hyoid bone.^25^, ^30^, ^49^, ^52^, ^53^ These measurements, however, rely on mandible position, which may rotate backwards during the normal course of orthodontic treatment.^62^ The use of a stable horizontal or vertical reference plane^26^, ^50^ or an independent landmark unaffected by orthodontic treatment^51^ would provide more accurate changes in hyoid bone positions.

#### 4.2.3 Changes in relation to incisor retraction

Airway dimensional change was reported to be correlated to upper incisor retraction distance by two studies, 26, 54 but amount of lower incisor retraction was not measured in these studies. For studies that included both upper and lower incisor measurements,^25^, ^52^ results revealed only a correlation with lower incisor retraction distance. On the other hand, no correlation to upper or lower incisor movement was also reported.^50^ It was believed that since maxillary incisors are located above the mandibular incisor, retraction of upper teeth is not expected to affect pharyngeal airway significantly compared with the retraction of the mandibular incisor.^50^ However, this inference has not been validated by research and requires investigation. In addition, the effect on airway dimensions may be independent of incisor inclination change.^50^

#### 4.2.4 Long‐term changes

All the studies lacked long‐term follow‐up. Partial reversion of the hyoid bone position and partial re‐establishment of airway dimensions twelve months after posterior surgical setback of the mandible has been reported.^63^, ^64^ Whether the same effect exists in orthodontic extraction cases is still unknown.

#### 4.2.5 Individual variability

Standard deviations often exceeded the magnitude of mean A‐P linear changes in East Asian lateral cephalometric studies, suggesting that A‐P dimensional reduction from incisor retraction was highly inconsistent. Standard deviations exceeding mean effect size was also found in South Asian studies^52^ and in hyoid bone movements measured on lateral cephalograms.^25^, ^50^ Wang et al^25^ highlighted a case where the retroglossal and hypopharyngeal linear dimensions decreased by 33.3% and 21.7%, respectively, far larger than the mean reduction in the study population. This suggests that some patients are more prone to airway diminution due to individual susceptibility and adaptability, and could be related to variance in oropharyngeal soft tissue factors.^50^, ^65^ Conversely, Keum et al^50^ noted that 15.15% of patients had a paradoxical increase in the retropalatal airway dimension after incisor retraction, compared with the mean decrease experienced by the study population. This implies that some patients are more resistant to airway diminution. Although East Asians, as a group, appear to experience a decrease in airway A‐P linear dimension at the oropharynx and hypopharynx, a modest proportion of patients may be entirely unaffected or may even experience an increase in airway dimension.

#### 4.2.6 Comparison of lateral cephalogram and computed tomography

The results of CT studies appear to show a larger percentage airway reduction with smaller individual variation. Although lateral cephalometric airway measurements have been reported to be reliable,^59^ 2D radiographs may not accurately reflect the 3D structure of the airway.^66^, ^67^, ^68^, ^69^ The semi‐automated quantitative software assessment of the airway may minimise measurement errors on CT, and oropharyngeal airway volume measurements on CT have excellent reliability.^70^ Furthermore, Zhang et al’s^49^ finding of compensatory lateral airway widening with A‐P narrowing in hyperdivergent skeletal Class II patients would not have been detectable without CT imaging. Airway dimension changes are therefore better assessed using CT than lateral cephalograms.

### 4.3 Changes in functional breathing

Almost all the studies measured only morphological changes. As studies about post‐orthodontic airway narrowing are primarily concerned with an increase in OSA predisposition, the use of morphological change as a surrogate for respiratory function is not ideal.^31^ Although a close relationship between pharyngeal narrowing, hyoid bone position and OSA has been reported,^14^, ^71^ airway narrowing may not uniformly increase predisposition to OSA for all patients as functional and non‐anatomic aetiologies are an important factor in up to 56% of OSA cases.^9^, ^10^

Polysomnography (PSG) is the diagnostic reference standard for OSA, but it is impractical to perform pre‐ and post‐orthodontic PSG due to access limitations.^72^ Functional breathing is more closely associated with OSA severity than morphologic changes^73^ and could be used as a substitute for PSG. However, only one study performed a simulated functional assessment of breathing.^54^ The lack of functional assessment was a flaw pointed out in an earlier review by Hu et al^31^ which remains unaddressed by the majority of studies included in the current review.

### 4.4 Geographic and racial differences

Although geographic region was not specified in the search protocol, all studies meeting the criteria originated from Asia. This may be attributed to the fact that orthodontic extractions are more common in Asian populations.^20^, ^74^ In addition, East Asian populations present more frequently with bimaxillary proclination and lip protrusion^75^, ^76^ that is orthodontically treated with premolar extractions and incisor retraction.^77^, ^78^ The results of the search suggest that orthodontic extractions for incisor retraction may be more frequently indicated in Asia compared with other geographic regions.

Based on the results of this review, East Asians may be particularly prone to airway narrowing and inferior hyoid movement from incisor retraction. This could be due to anatomical characteristics of East Asians. Decreased cranial base dimensions in East Asians may have important implications in the pathogenesis of OSA.^79^ Chinese patients have greater craniofacial bony restriction and lower obesity and BMI when compared to Caucasians with the same degree of OSA or sleep‐disordered breathing (SDB).^3^, ^80^, ^81^, ^82^ Aside from craniofacial characteristics, OSA predisposition could also be related to the higher percentage of body fat for an equivalent level of BMI in Asian populations^83^, ^84^ compared with non‐Asian ones. Comparing between Asian subjects, Chinese were found to have greater odds for moderate to severe SDB than Indians after adjustment for age, sex and BMI.^5^ As bimaxillary protrusion patients have been found to have greater mouth breathing habits,^75^ larger tongue size^75^, ^85^ and greater soft palate thickness and length,^86^ the high prevalence of bimaxillary proclination in East Asians^75^, ^76^ could be construed as an adaptive trait to the inherent anatomic congestion. It is thus not surprising for East Asians to be prone to airway narrowing from orthodontic extractions.

Although a recent American white paper has posited that orthodontic extractions do not impact airway size or risk of OSA,^32^ the results of this review show that:

1. Indications for orthodontic extractions in Asian populations are different from other geographic regions. Extractions for incisor retraction may be more commonly indicated in Asian populations.
2. Different ethnicities may have different airway responses to incisor retraction.

Geographic differences would also account for the contradictory results on AHI and OSA prevalence after orthodontic extraction treatment reported by Fukuda et al^18^ and Larsen et al,^19^ as one study was conducted in Japan and the other in America.

### 4.5 Limitations and future work

#### 4.5.1 Review level

The review was restricted to studies published in the English language and is therefore subject to language and possibly publication bias.^87^ The inclusion of non‐English language studies may, however, not significantly change the results of this systematic review.^88^, ^89^, ^90^, ^91^ From the preliminary literature review, the decision was made *a priori* not to limit the types of clinical studies. The majority of the clinical studies on this topic were case series, before–after studies and other uncontrolled or poorly controlled observational study designs. Uncontrolled before–after studies are deemed as case series by the Cochrane network^92^, ^93^ and at risk of bias,^94^, ^95^ but can provide sufficient information to calculate treatment effects, although not relative risk.^96^

#### 4.5.2 Study level

All selected studies were uncontrolled and observational in design and were at increased risk of bias.^95^ All but two^26^, ^54^ of the studies were retrospective. Most of the studies did not report demographic data such as ethnicity and race in detail, but national and city‐based population census data show that most of the studies come from highly racially homogenous populations, representative of the population concerned. Reporting of clinical information and outcomes was also generally poor, with multiple errors, incomplete data and conclusions that were incongruent with reported data. While some clarifications were received from corresponding authors, not all authors responded. Given the small treatment effect, large individual variation in airway response observed in lateral cephalometric studies and the lack of control groups, the decrease in airway dimension cannot be confidently attributed to the intervention, especially since case series and uncontrolled studies are prone to overestimation of effects.^97^ The use of untreated or non‐extraction controls imaged at pre‐ and post‐treatment time points, matched for age, gender, race, skeletal profile and weight changes, would have mitigated this limitation.

#### 4.5.3 Recommendations for future work

Learning from the inadequacies of prior studies, future research in this area should incorporate:

1. Detailed reporting of racial demographics, age, growth status, gender, horizontal and vertical skeletal subtypes, gender, oropharyngeal soft tissues and other possible confounders, as well as the intervention received, such as extraction pattern.
2. CT imaging for airway assessment.
3. Use of stable reference points for hyoid positional change assessment.
4. Functional assessment of breathing including polysomnography.
5. Use of untreated or non‐extraction matched controls imaged at pre‐ and post‐treatment.
6. Appraisal of both upper and lower incisor changes and correlating this to airway dimensional changes.
7. Long‐term follow‐up to monitor for adaptive reversions of airway dimensions.

## 5 CONCLUSIONS

Within the limitations of this systematic review, the following conclusions could be made:

1. Linear airway response to incisor retraction measured on lateral cephalograms varied substantially, while linear, cross‐sectional and volumetric measurements of posterior airway space using CT showed larger effect sizes and smaller variations, providing stronger evidence of airway narrowing with bicuspid extractions and incisor retraction.
2. Hyoid bone positional changes in response to bicuspid extractions and incisor retraction varied substantially.
3. Functional breathing response to bicuspid extractions and incisor retraction was not adequately studied.
4. Orthodontic extractions for incisor retraction may be more frequently indicated in Asia, and East Asians seem particularly susceptible to airway narrowing and postero‐inferior hyoid movement with bicuspid extractions and incisor retraction.
5. Better designed CT studies are needed before definitive conclusions can be drawn due to small effect size and large variability.

## CONFLICT OF INTEREST

The authors have stated explicitly that there are no conflicts of interest in connection with this article.
